# Study protocol: a single-blind, multi-center, randomized controlled trial comparing dynamic intraligamentary stabilization, internal brace ligament augmentation and reconstruction in individuals with an acute anterior cruciate ligament rupture: LIBRƎ study

**DOI:** 10.1186/s12891-019-2926-0

**Published:** 2019-11-18

**Authors:** Christiaan H. W. Heusdens, Katja Zazulia, Ella Roelant, Lieven Dossche, Damien van Tiggelen, Johan Roeykens, Elke Smits, Johan Vanlauwe, Pieter Van Dyck

**Affiliations:** 1 Department of Orthopaedics, Antwerp University Hospital, Wilrijkstraat 10, 2650 Edegem, Belgium; 2 Clinical Trial Center (CTC), CRC Antwerp, Antwerp University Hospital, University of Antwerp, Wilrijkstraat 10, 2650 Edegem, Belgium; 3 Department of Traumatology and Rehabilitation, Queen Astrid Military Hospital, Bruynstraat 1, 1120 Neder-Over-Heembeek, Belgium; 4 Department of Rehabilitation, Antwerp University Hospital, Wilrijkstraat 10, 2650 Edegem, Belgium; 5 Department of Medical management, Antwerp University Hospital, Wilrijkstraat 10, 2650 Edegem, Belgium; 6 Department of Orthopaedics and Traumatology, University Hospital Brussels, Laarbeeklaan 101, 1090 Jette, Belgium; 7 Department of Radiology, Antwerp University Hospital, Wilrijkstraat 10, 2650 Edegem, Belgium

**Keywords:** Orthopaedics, Knee, Anterior cruciate ligament, Acute anterior cruciate ligament rupture, Anterior cruciate ligament reconstruction, Anterior cruciate ligament repair, Anterior cruciate ligament injury

## Abstract

**Background:**

The current gold standard for the treatment of an anterior cruciate ligament (ACL) rupture is reconstruction with tendon graft. Recently, two surgical ACL repair techniques have been developed for treating an acute ACL rupture: Dynamic Intraligamentary Stabilization (DIS, Ligamys®) and Internal Brace Ligament Augmentation (IBLA, *Internal*Brace™). We will conduct a single-blind, multi-center, randomized controlled trial which compares DIS, IBLA and reconstruction for relative clinical efficacy and economic benefit.

**Methods:**

Subjects, aged 18–50 years, with a proximal, primary and repairable ACL rupture will be included. DIS is preferably performed within 4 weeks post-rupture, IBLA within 12 weeks and reconstruction after 4 weeks post-rupture. Patients are included in study 1 if they present within 0–4 weeks post-rupture and surgery is feasible within 4 weeks post-rupture. Patients of study 1 will be randomized to either DIS or IBLA. Patients are included in study 2 if they present after 4 weeks post-rupture and surgery is feasible between 5 and 12 weeks post-rupture. Patients of study 2 will be randomized to either IBLA or reconstruction. A total of 96 patients will be included, with 48 patients per study and 24 patients per study arm. Patients will be followed-up for 2 years. The primary outcome is change from baseline (pre-rupture) in International Knee Documentation Committee score to 6 months post-operatively. The main secondary outcomes are the EQ-5D-5 L, Tegner score, Lysholm score, Lachman test, isokinetic and proprioceptive measurements, magnetic resonance imaging outcome, return to work and sports, and re-rupture/failure rates. The statistical analysis will be based on the intention-to-treat principle. The economic impact of the surgery techniques will be evaluated by the cost-utility analysis. The LIBRƎ study is to be conducted between 2018 and 2022.

**Discussion:**

This LIBRƎ study protocol is the first study to compare DIS, IBLA and ACL reconstruction for relative clinical efficacy and economic benefit. The outcomes of this study will provide data which could aid orthopaedic surgeons to choose between the different treatment options for the surgical treatment of an acute ACL rupture.

**Trial registration:**

This study is registered at ClinicalTrials.gov; NCT03441295. Date registered 13.02.2018.

## Background

The anterior cruciate ligament (ACL) is an important stabilizer of the knee. The ACL prevents anterior tibial translation and provides constraint to tibial internal rotation [1]. 2.5% of the ACL consists of proprioceptors [2, 3], which give feedback to the brain and spinal cord about the positioning of the knee joint. These proprioceptors play a role in defining and controlling normal joint movement [4]. Abnormal range or speed movements of the joint will trigger the brain to stimulate appropriate musculature to stabilize the joint.

Injury to the ACL is the most common ligament injury of the knee joint. Ruptures of the ACL mainly occur in young people (aged 16–40 years) performing pivoting sports like football, hockey, basketball and skiing. Each year 0.03 to 1.62% of amateur athletes and 0.15 to 3.67% of professional athletes are affected [5]. In Belgium, 6.745 cruciate ligament surgeries were performed in 2017 (according to RIZIV/INAMI, Belgian National Sickness and Invalidity Insurance Institute). The socio-economic burden is considerable as the majority of ACL injuries occur in people of working age. The indirect costs related to absence from work, school or university are in addition to costs borne by the healthcare system [6].

Since the mid-eighties the gold standard for an operative treatment of an ACL rupture is reconstruction with a tendon graft. This involves removing native ACL tissue including its proprioceptors. The ligament is often replaced with autograft donor tendon(s), such as a hamstrings tendon or a part of the patellar tendon. A number of problems have been identified as graft harvest is associated with a degree of morbidity from tissue loss. Hamstrings muscle weakness following harvesting averages 10% in most studies [7]. Revascularization of the graft takes 6–12 months and ingrowth of the graft in the bone takes up to 2 years [8]. Another disadvantage of conventional ACL reconstruction is the rather long period of revalidation associated with a huge socio-economic burden. A successful recovery from an ACL reconstruction encompasses intensive physiotherapy and requires a lot of effort, dedication, time and perseverance. According to Ardern et al. [9], the return to competitive sports after ACL reconstruction is only 44 to 55%. In another study, Biau et al. [10] found that only 40% of patients gain full functional recovery. Nagelli and Hewett postulated that delay in returning to sports for the first 2 years will significantly reduce the incidence of second ACL injuries [11]. Given the limitations and risks associated with the current gold standard treatment of an ACL rupture, there is room for improvement.

The last few years there is a renewed interest in ACL repair as an alternative operative treatment for the acute ruptured ACL. Two novel surgical ACL repair techniques have been developed and proof of concept has been established for treating an acute ACL rupture: Dynamic Intraligamentary Stabilization (DIS, Ligamys®, Mathys Ltd., Bettlach, Switzerland) and Internal Brace Ligament Augmentation (IBLA, *Internal*Brace™, Arthrex GmbH, Naples, Florida) repair techniques [12, 13].

The DIS technique has been shown to successfully induce self-healing of a ruptured ACL in animal models. Biomechanical studies in human cadaveric knees have shown that DIS restores knee joint kinematics comparable to that of an ACL-intact knee and provide further evidence that DIS is capable of providing knee joint stability during ACL healing [14–16]. A clinical experience of the first 3 years after DIS in a large case series was reported by Henle et al. [17]. They found that anatomical repositioning, along with DIS and micro-fracturing, leads to clinically stable healing of the ruptured ACL in 96% of patients. In their study, most patients exhibited a normal knee function, reported excellent satisfaction, and were able to return to their previous levels of sports activities. The same group also presented excellent outcomes and satisfaction with regards to the treatment result of all the patients with a functionally healed ACL with a 5-year follow-up [18]. Factors influencing the success of ACL repair with DIS were described by Krismer et al. and focus on patient selection [19]. In general, a higher percentage of successful outcomes after ACL repair are seen in patients with an acute, proximal ACL rupture because these tend to have better healing capacity and tissue quality. Although no significant differences were found in treatment costs and revision rates, patients treated by DIS benefited from nearly 1 month shorter absence from work as compared to patients treated by conventional ACL reconstruction. This was possibly due to the fact that the DIS procedure is recommended up to the first 21 days after ACL injury. For an ACL reconstruction there is no time limit [20].

Relatively little data is available in the literature on the IBLA technique. The proof of concept was provided by Mackay et al. [21]. They reported clinical outcomes at a minimum of 1 year follow-up, and found that IBLA is at least as effective in restoring stability and function of the knee as the conventional ACL reconstruction, with the greatest improvements seen in the decreased time of recovery. The same group also presented good functional outcomes along with radiographic and arthroscopic evidence of a healed ACL, in one of the first patients treated with IBLA [22]. In a 2 year follow-up of 42 patients treated with the IBLA technique, two patients (4.8%) reported an ACL re-rupture. Heusdens et al. conclude that repair with this technique could be clinically relevant as a treatment option for patients with an acute, proximal ACL rupture which is not retracted and of good tissue quality [23]. In addition, Smith et al. demonstrated the potential for excellent outcomes for paediatric ACL repair with temporary IBLA as an attractive alternative to ACL reconstruction [24].

To our knowledge, there is only one randomized controlled trial (RCT) published comparing an ACL repair technique with the conventional ACL reconstruction [25]. Hoogeslag et al. concluded that DIS is not inferior to ACL reconstruction in terms of subjective patient-reported outcomes 2 years postoperatively, but for reasons other than revision ACL surgery due to re-rupture a higher number of related adverse events were seen in the DIS group [25].

Furthermore, there are no RCTs published comparing different ACL repair techniques with each other. Because of the lack of RCTs orthopaedic surgeons are careful in adapting these new repair techniques. This clinical trial was designed in order to provide more scientific evidence on which of the three surgery techniques could possibly be the most clinically and economically effective for treating an acute ACL rupture.

## Methods

### Study objectives

#### Primary objective

To determine the clinical efficacy of two alternative techniques DIS and IBLA in comparison to the conventional ACL reconstruction for treating an acute ACL rupture.

We are interested in the three pairwise comparisons between the techniques: DIS versus ACL reconstruction, IBLA versus ACL reconstruction and DIS versus IBLA.

#### Secondary objectives

- To assess whether DIS and IBLA offer an improvement in quality of life, patient satisfaction and functioning of the patient compared to conventional ACL reconstruction.
- To assess whether DIS and IBLA result in a shorter recovery period compared to conventional ACL reconstruction in terms of the mobilization period with crutches after the surgery, and return to work and sports period.
- To assess whether there is a difference between DIS and IBLA versus conventional ACL reconstruction in terms of pain and complications during and after surgery.
- To assess whether there is a difference between DIS and IBLA versus conventional ACL reconstruction in terms of ACL re-ruptures/failures.
- To determine the economic benefit of the two alternative techniques DIS and IBLA in comparison to the conventional ACL reconstruction for treating an acute ACL rupture.
- For all of these objectives also the mutual comparison between DIS and IBLA will be considered.

### Study design

The **LIBRƎ** trial is a single-blind, multi-center, prospective, RCT comparing **L**igamys® anterior cruciate ligament (ACL) repair, ***I****nternal***B**race™ ACL repair and conventional ACL **re**construction for relative clinical efficacy and economic benefit.

Patients admitted for an acute traumatic knee sprain needing ACL surgery will be recruited at the Antwerp University Hospital and University Hospital Brussels, Belgium between February 2018 and June 2020.

The LIBRƎ study is single-blind, meaning that the patient and the physiotherapist are blinded to the performed surgical technique, but they are aware in which part of the study the patient participates (study 1 or study 2). Patient’s physiotherapist will follow a study-specific rehabilitation protocol (identical for the three treatment arms), depending on the readiness of the patient for a next phase and not restricted to a predefined time line.

**Fig. 1:**
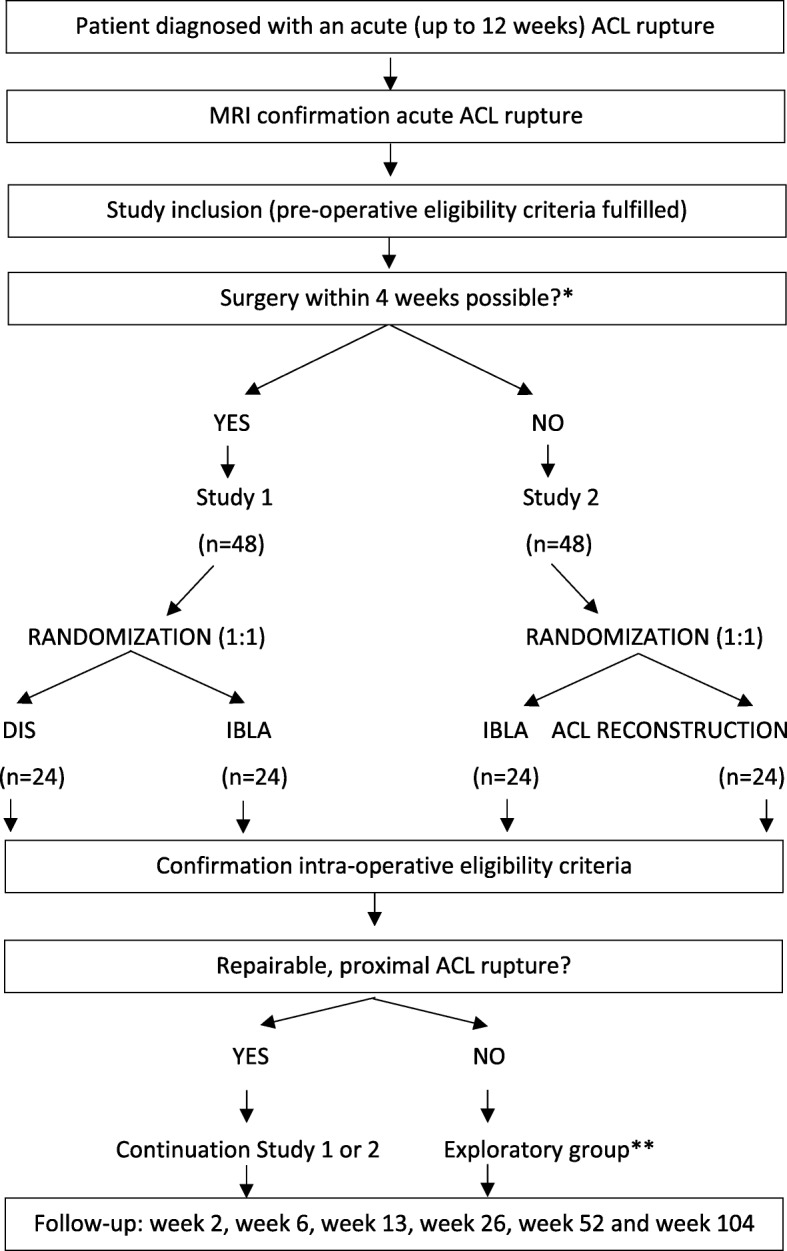
Study flow diagram: screening, inclusion, surgery and follow-up. *Depends on when the patient presents at the consultation desk after the ACL rupture. **Patients in the exploratory group will be replaced, but are still part of the study. ACL = Anterior Cruciate Ligament, DIS = Dynamic Intraligamentary Stabilization, IBLA = Internal Brace Ligament Augmentation, MRI = Magnetic Resonance Imaging

In the study design, the time-dependent nature of ACL repair surgeries has been taken into account. The company Mathys Ltd. advices DIS to be performed within 3 weeks after the ACL rupture. Previous ACL repair surgery experience from the authors (CH and LD) led to a prolonged DIS ACL repair period within 4 weeks post-rupture. IBLA can be performed up to 12 weeks after the ACL rupture. ACL reconstruction is preferably performed when the knee has regained its function. This is commonly after 4 weeks post-rupture and can be performed up to many years later. Taken into account these time limits there will be two parallel studies (flowchart, Fig. 1):

- Study 1 (RCT 1) with a time frame 0–4 weeks post-rupture: patients are randomized 1:1 to DIS or IBLA
- Study 2 (RCT 2) with a time frame 5–12 weeks post-rupture: patients are randomized 1:1 to IBLA or conventional ACL reconstruction.

Allocation to either study 1 or study 2 depends on the patient’s admission time point after ACL injury and if it is practically feasible to perform ACL surgery within 4 weeks. There is a patient and referral delay, a magnetic resonance imaging (MRI) needs to be performed to confirm the ACL rupture and the surgery has to be planned. On the other hand, inclusion and surgery between week 5–12 after injury is expected to be more manageable to plan. There will be an intra-operative confirmation in study 1 and study 2 of the ACL’s eligibility for repair. If the proximal ACL rupture is not suitable for repair, ACL reconstruction will be performed. To keep the three arms (DIS, IBLA and the conventional reconstruction arm) comparable, patients with a non-repairable proximal ACL rupture will be replaced by a new patient even if they were randomized to the conventional ACL reconstruction. These patients will be seen as excluded from the randomized medical device (but not excluded from the study) and will undergo a conventional ACL reconstruction (exploratory group). These patients will be treated as a sub-population and will not be included in the primary analysis.

### Study population

#### Inclusion criteria

**Fig. 2:**
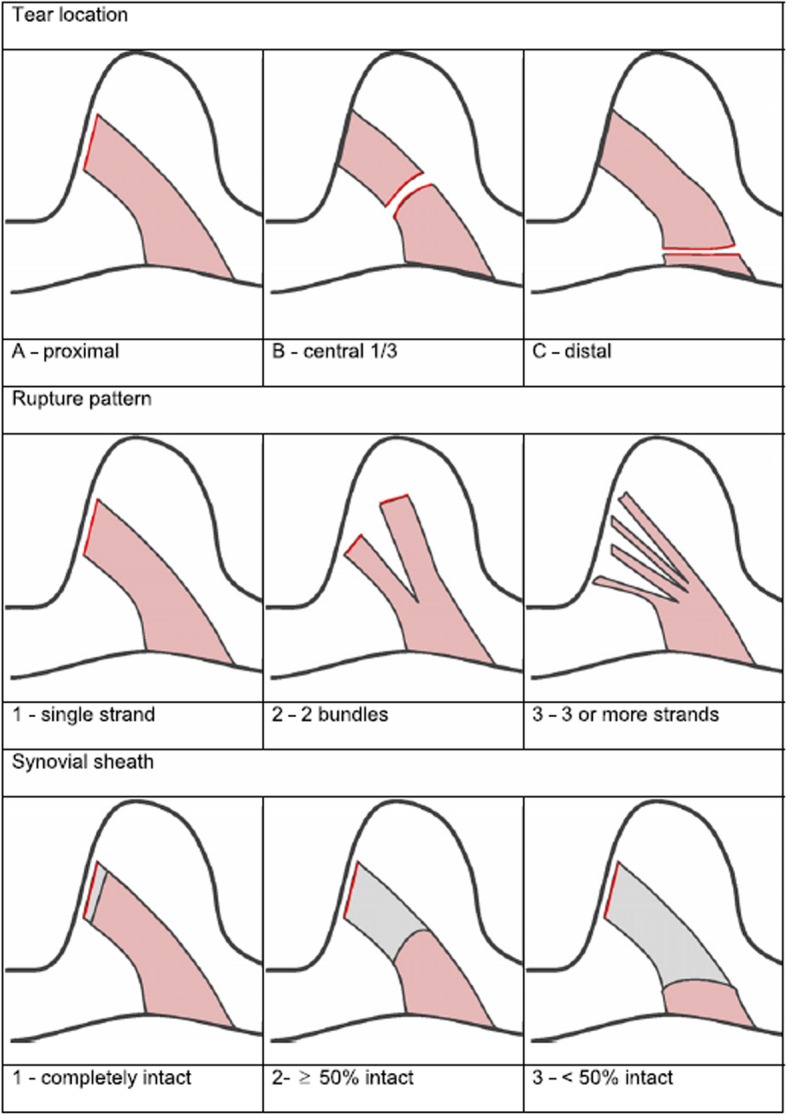
Three-digit ACL rupture classification. The first digit describes the ACL rupture location: ‘A’ for proximal third, ‘B’ for central third and ‘C’ for distal third. The second digit is the ACL rupture status: ‘1’ for 1 bundle, ‘2’ for 2 bundles and ‘3’ for multilacerated. The third digit describes the ACL synovial tube: ‘1’ for completely intact, ‘2’ for ≥50% intact and ‘3’ for < 50% intact [17]

- Primary acute proximal ACL rupture: 3-digit ACL rupture classification, type A (MRI and intra-operative confirmation) [17] (Fig. 2).
- Age: 18–50 years.
- Presented and planned surgery within 4 weeks after the ACL rupture (Study 1).
- Presented and planned surgery between 5 and 12 weeks after the ACL rupture (Study 2).
- The ACL remnant is suitable for repair in the three treatment arms: at least 75% of the distal ACL remnant must be in contact with the proximal remnant/femoral condyle (intra-operative confirmation).
- Mentally and verbally capable of participating in the study.
- Written informed consent (according to the ICH-GCP Guidelines).

#### Exclusion criteria

- Posterior cruciate ligament injury and/or posterolateral ligamentous complex grade 3 injury, lateral collateral ligament grade 3 injury or medial collateral ligament grade 3 injury.
- Osseous fractures or trauma that could impair rehabilitation and/or ACL repair.
- Neurological disorder or systemic disease.
- Inflammatory disease, rheumatoid arthritis, spondyloarthropathy or active malignancy
- Non-sportive: Tegner score of less than 3.
- Not suited for intervention due to lack of mobility, meaning not achieving 90° of flexion before surgery.

### Study intervention

#### Conventional ACL reconstruction

**Fig. 3:**
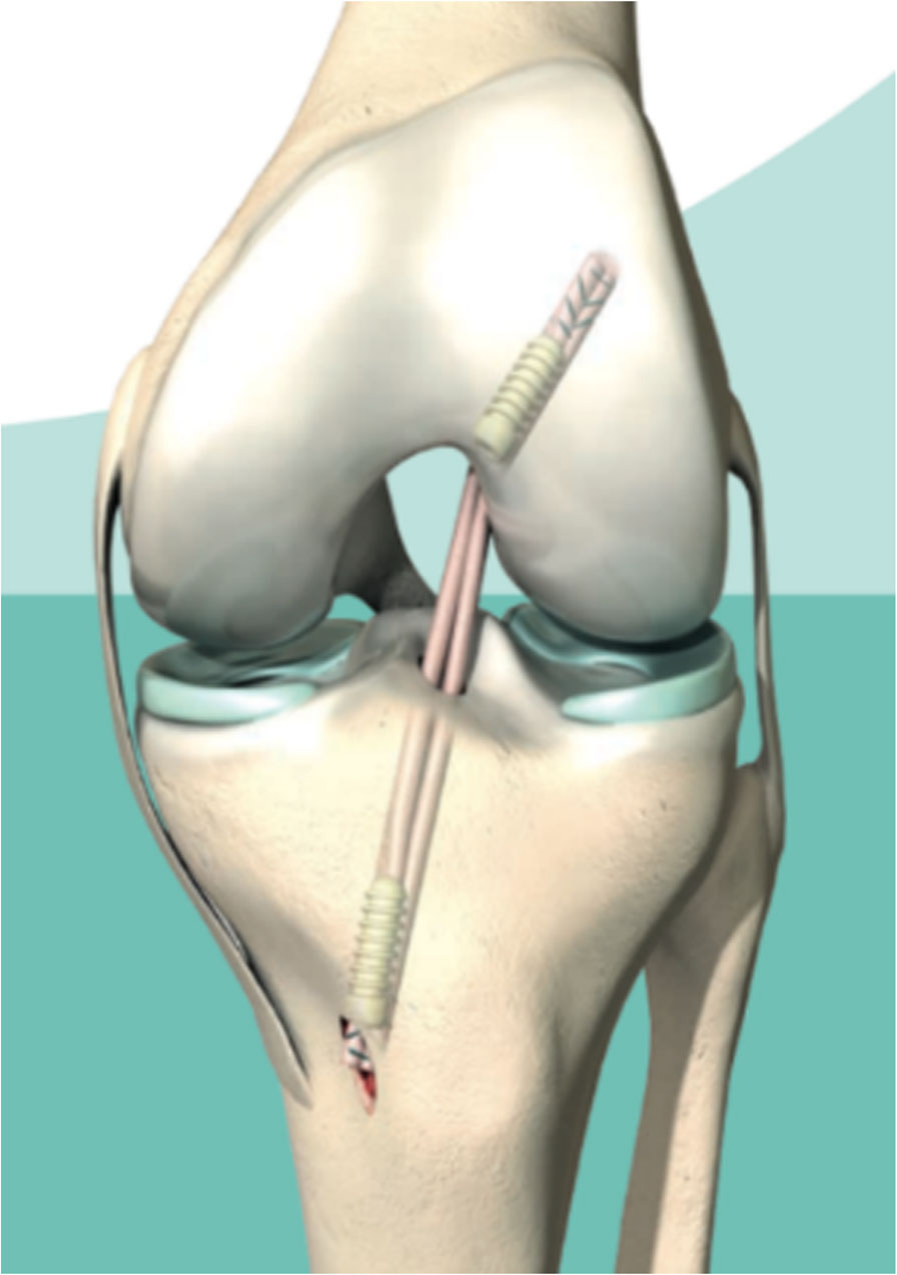
ACL reconstruction, left knee, frontal view,©Mathys Ltd. Bettlach. Permission was granted by the company©Mathys Ltd. Bettlach to use this picture in a journal article

ACL reconstruction involves replacing the torn ligament with a harvested graft (quadrupled semitendinosus) taken from the patient’s ipsilateral knee (Fig. 3) [26]. This arthroscopic procedure involves harvesting the hamstrings tendon graft, removing the ruptured ACL and drilling tunnels in the femur and tibia to place the graft in the same anatomical position as the native ACL. The graft is fixed on the femur with an adjustable suture button, and on the tibia with a post- and interference screw.

#### DIS

**Fig. 4:**
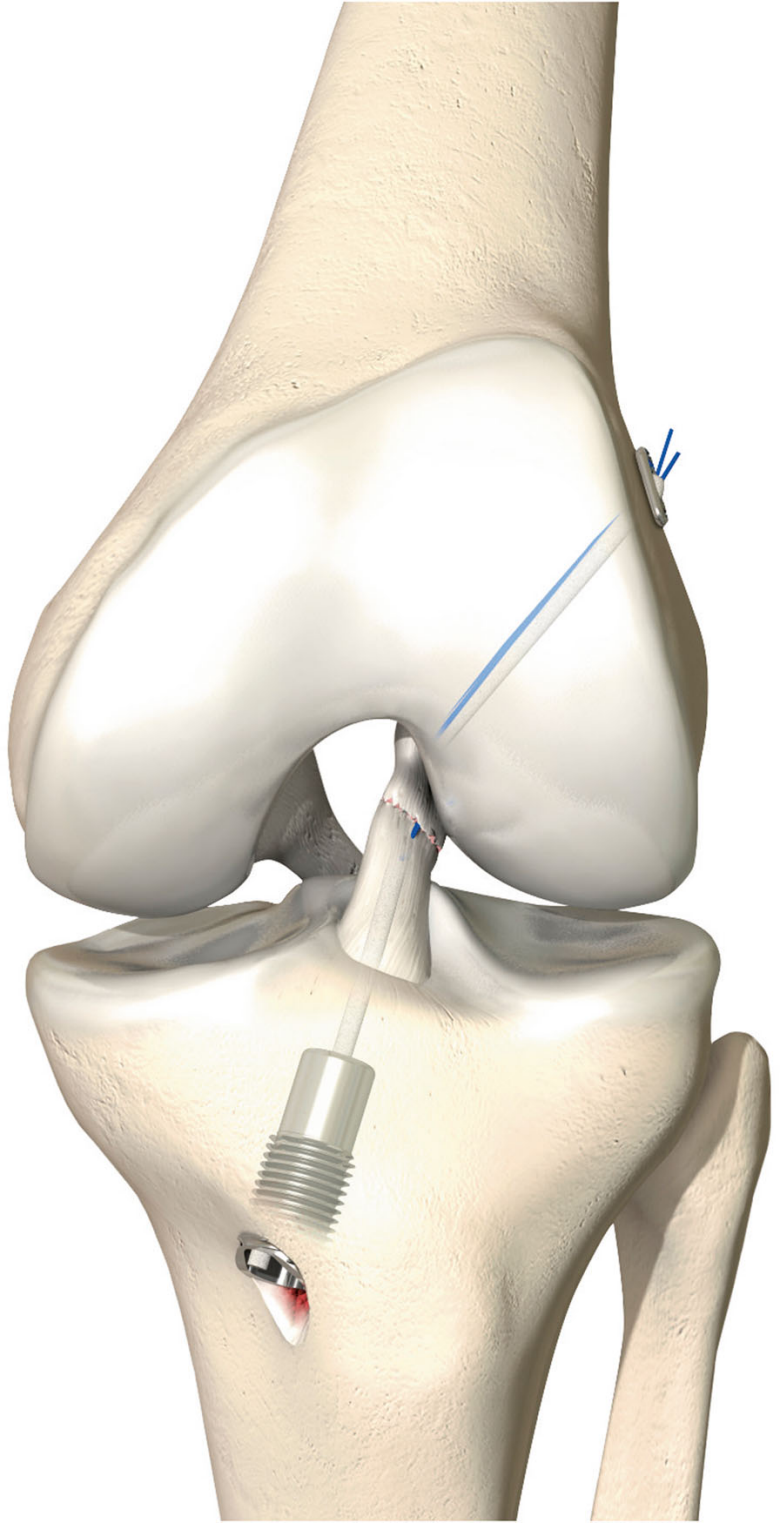
Ligamys® technique, left knee, frontal view. Picture can be found in the ‘Ligamys® Surgical technique’ brochure,©Mathys Ltd. Bettlach. Permission was granted by the company©Mathys Ltd. Bettlach to use this picture in a journal article

The DIS repair technique was described by Eggli et al. (Fig. 4) [12]. The tibial stump of the ACL is reduced to the femoral footprint by transosseous sutures thereby restoring the anatomical position of the ACL [15]. Additional to the technique described by Eggli et al., lasso sutures will be used to reduce the tibial stump, if the stump cannot be reduced with PDS sutures. With this adaption, multi-bundle ruptures can be repaired. The knee is stabilized with a strong polyethylene cord which is passed on the tibial side behind the tibial footprint to prevent damage to the ACL tibial blood and nerve supply. At the femoral side the cord is passed through the anatomical footprint after carrying out micro-fracturing at this site. Using a dynamic spring-screw implant, the cord is brought under tension on the anteromedial aspect of the tibia just above the pes anserinus insertion. The proximal tibia is hereby pulled in a constant posterior drawer position with a force of 50–80 Newton depending on the sex of the patient. It is hypothesized that DIS repair continually stabilizes the knee and therefore can enable mechanically stable ACL healing [12].

#### IBLA

**Fig. 5:**
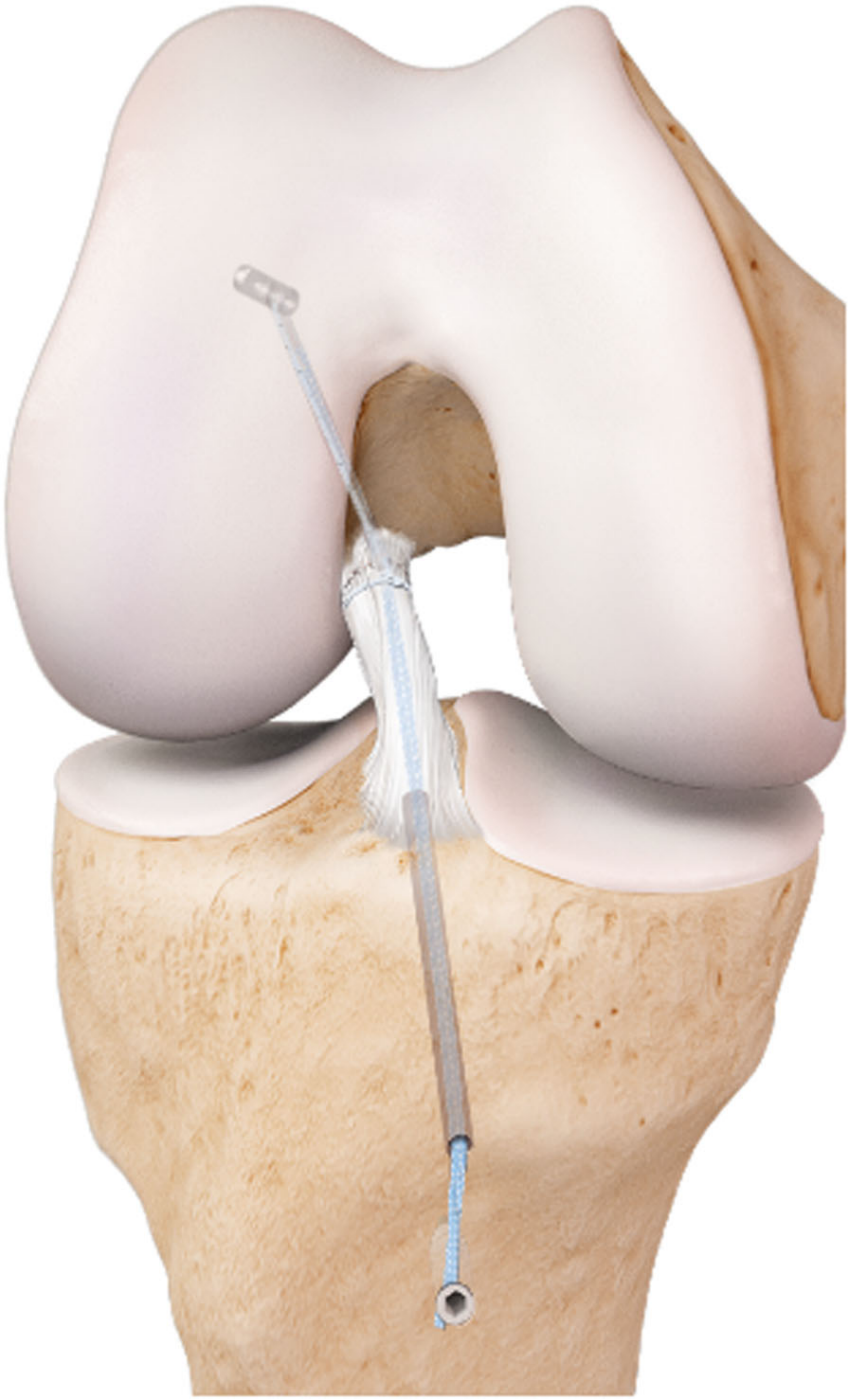
*Internal*Brace™ technique, left knee, frontal view. Picture can be found in the ‘ACL Primary Repair with *Internal*Brace™ Surgical technique’ brochure,©Arthrex GmbH. Permission was granted by the company©Arthrex GmbH to use this picture in a journal article

The IBLA repair technique involves repair of the ACL as well (Fig. 5) [13, 27]. The proximal end of the ACL stump is re-approximated against the medial wall of the lateral femoral condyle or the proximal remnant with a lasso suture. The knee is stabilized with a high strength tape, fixed on the femur with a femoral button, passing besides or through the ACL from the femoral footprint to the tibial footprint and fixed on the tibia with a bone anchor. The *Internal*Brace™ reinforces the ligament as a secondary static stabilizer, encouraging natural healing of the ligament by protecting it during the healing phase and supporting early mobilization.

The three surgical techniques will be performed in Antwerp University Hospital and University Hospital Brussels using similar surgical protocols. No additional anterolateral procedure will be performed. In case of a non-repairable ACL rupture, a conventional ACL reconstruction will be performed. In case of failure/re-rupture of the repaired ACL (DIS/IBLA technique) a conventional ACL reconstruction can be performed. In case of failure/re-rupture of the reconstructed ACL, a revision of the reconstruction can be performed.

### Study endpoints

#### Primary endpoint

The primary endpoint is change in International Knee Documentation Committee (IKDC) from baseline (pre-rupture) to 6 months post-operatively. The difference in IKDC score between the reconstruction technique and the repair techniques (DIS/IBLA) is expected to be the largest at 6 months postoperatively, due to the proprioceptive conservation with the repair techniques.

The IKDC is a commonly used instrument to determine the outcome following various knee procedures, including ACL reconstructions [28]. In essence, it is a subjective well-known tool that provides patients with an overall function score (range 0–100). The score is interpreted as a measure of function with higher scores representing higher levels of function. The questionnaire addresses three categories: symptoms (pain, swelling, stiffness, etc.), activity (rising from chair, going up and down stairs, jumping, squatting, etc.) and knee function [29].

#### Secondary endpoints

- Pain experienced by the patients as measured by Visual Analogue Scale (VAS) pain score [30, 31].
- Overall functioning by the patient, related to the knee, as measured by Tegner and Lysholm score [32].
- Health-Related Quality of Life (HRQoL) as measured by EQ-5D-5 L [33, 34].
- Time to recovery as measured by mobilization with crutches, return to work and sports.
- Patient satisfaction towards the surgery and revalidation level as measured by VAS satisfaction.
- Mechanic functioning of the knee as measured by Lachman test; single leg hop, triple leg hop, triple crossover hop and drop jump test; proprioceptive measurement; isokinetic measurement; and knee function [35–42].
- Intra- and post-operative complications: adverse device effects (ADEs) and serious adverse events (SAEs).
- Success of the operation techniques defined as the number of non-failures.
  - A failure is defined as:
    - A re-rupture of the graft or the repaired ACL: clinically and MRI-confirmed
    - Instability complaints: AP translation difference of more than 3 mm (Lachman test) between the injured knee and the contralateral knee, and subjective instability complaints [17].
- The number of physiotherapy and (extra) orthopaedic surgeon consults.
- ACL healing will be evaluated with MRI [43].

### Study procedures

Table 1 gives an overview of the clinical and radiological evaluations the patient will encounter in both centers following written informed consent. The physiotherapy will be performed by the patient’s physiotherapist according to the study-specific rehabilitation protocol. This physiotherapy protocol is based on five phases. Proceeding to the next phase depends on the patients rehabilitation progress and not on the post-operative time.

**Table 1:** Pre-, intra- and post-operative clinical and radiological evaluations

Timepoint	Clinical and radiological evaluations
Pre-operative	Pre-rupture: Tegner score, Lysholm score and IKDC score Post-rupture: VAS pain, knee function, Lachman test, EQ-5D-5 L, isokinetic and proprioceptive measurement, and MRI
Intra-operative	Knee function, pivot shift test, surgery time and complications
Post-Operative	
Day 1	VAS pain, return to work rupture-surgery interval, EQ-5D-5 L and complications
Week 2	VAS pain, return to work, knee function, EQ-5D-5 L and complications
Week 6	VAS pain and satisfaction, mobilization with crutches, return to work, return to sports, knee function, Lachman test, EQ-5D-5 L, complications and X-ray knee
Week 13 (3 months)	VAS pain and satisfaction, mobilization with crutches, return to work, return to sports, knee function, Lachman test, EQ-5D-5 L, Tegner score, Lysholm score, IKDC score, complications, and isokinetic and proprioceptive measurement
Week 26 (6 months)	VAS pain and satisfaction, return to work, return to sports, knee function, Lachman test, EQ-5D-5 L, Tegner score, Lysholm score, IKDC score, complications, number of physiotherapy and orthopaedic surgeon consults, single leg and triple leg hop test, isokinetic and proprioceptive measurement, and MRI
Week 52 (1 year)	VAS pain and satisfaction, return to work, return to sports, knee function, Lachman test, EQ-5D-5 L, Tegner score, Lysholm score, IKDC score, complications, number of physiotherapy and orthopaedic surgeon consults, single leg and triple leg hop test, triple crossover hop and drop jump test, isokinetic and proprioceptive measurement, and MRI
Week 104 (2 years)	VAS pain and satisfaction, return to work, return to sports, knee function, Lachman test, EQ-5D-5 L, Tegner score, Lysholm score, IKDC score, complications, number of physiotherapy and orthopaedic surgeon consults, and MRI

*IKDC* International Knee Documentation Committee, *MRI* magnetic resonance imaging, *VAS* visual analogue scale

MRI is performed on a 3 T system (Magnetom Prisma Tim, Siemens, Erlangen, Germany) for IBLA and ACL reconstruction arms. For patients treated by DIS, a 1.5 T system (Magnetom Aera Tim, Siemens, Erlangen, Germany) with metal artefact reduction sequences will be used. The 4-level Howell classification, originally proposed for grading ACL graft, is adapted for evaluating the repaired ligaments [44]. The morphology and signal intensity of the ACL graft or repair will be subjectively assessed on conventional MRI [45].

When re-surgery is needed or a failure occurs, the patient is not excluded from the study and the follow-up will continue according to this protocol. Under re-surgery, we consider all surgeries a patient undergoes on the side of the injured knee affecting his/her mobility. Re-surgery following re-rupture will be considered as a failure.

If the proximal ACL rupture is not suitable for repair, the patient will undergo an ACL reconstruction (exploratory group). These patients will be followed according to this protocol except for the post-operative MRI measurements on week 52 and 104, and the post-operative isokinetic measurements on week 13, 26 and 52 as these measurements are study-specific and not standard of care.

### Study duration

The start date of inclusion is February 2018, expected end date of inclusion is June 2020. The follow-up duration is 2 years. Last patient follow-up visit is expected in June 2022.

### Sample size

The primary outcome is change in IKDC score from baseline (pre-rupture) to 6 months post-operatively. A difference in IKDC change score of 13 points between the treatment arms is considered clinically meaningful as described by Irrgang et al. and Collins et al. [46, 47]. In order to detect an effect of 13 IKDC points with 80% statistical power between any of the two treatment arms using an independent samples t-test in which alpha is 0.025 (alpha corrected for the fact that per study data set 2 tests will be performed), assuming a standard deviation of 13 on the change in IKDC score per arm (SD found in pilot study), we need 21 patients per arm. Taking into account a drop out of 10%, we need 24 patients per arm or 48 patients in total which have to be randomized.

In study 1, 24 patients per arm (DIS and IBLA) will be included and in study 2, 24 patients per arm (IBLA and ACL reconstruction) will be included. The total number of patients to be included is 96. We expect that 50% of the patients will be included in Antwerp University Hospital and 50% in University Hospital Brussels. We also expect that the total number of addressed patients will range from 96 to 106, since we expect that about 10% of the patients will not have a repairable, proximal ACL rupture. They will not be eligible (any more) after the intra-operative confirmation and these patients (exploratory group) will not contribute the number of 96 patients needed for inclusion.

### Randomization and blinding

Randomization will take place when the patient is diagnosed with an acute ACL rupture, which is MRI confirmed, fits all the in- and exclusion criteria (expect for the two inclusion criteria which are intra-operatively confirmed: proximal and repairable ACL rupture), and when the patient has signed the informed consent. The randomization procedure will be generated in Castor Electronic Data Capture software (Castor EDC, Amsterdam, the Netherlands). Castor uses stratified block randomization. Randomization will be stratified according to center. Per center a permuted block randomization with variable block size will be used.

The LIBRƎ study is a single-blind study meaning that the treating physicians (principal and sub-investigators) and the study coordinator are not blinded. The patient and the patient’s physiotherapist are blinded.

### Statistical methods

#### Data management and analysis software

Castor EDC software will be used for data management. This ICH-GCP compliant web-based software covers all aspects of data management, data collection, ADE/SAE reporting, randomization, patient surveys and monitoring in multi-center studies. Patients will receive an e-mail via Castor with a request to fill-in the questionnaires.

All data is gathered from study 1 and 2 for data collection and cleaning via direct entry in Castor EDC (electronic case report forms (eCRF)). The eCRF data in the Castor EDC are stored on an accredited data center hosting in the Netherlands (accreditations: ISO 27001:2013, ISO 9001 and NEN7510). Data can be retrieved from this data center at any point in time to perform the required statistical patient data analyses led by Antwerp University Hospital. Full audit trail is available to log every change to the trial’s data as well as which user made the change.

All statistical analyses will be performed in SAS version 9.4 or higher, R version 3.3.2 or higher, or SPSS 24.

### Statistical analyses

#### Analysis of the primary outcome

To answer the research question comparing the three treatment arms the research question is split up in three separate questions:

- *Is there a difference between DIS and IBLA (≤4 weeks)?*

Results from study 1 will be used in a linear regression model with IKDC at 6 months as outcome and treatment, and IKDC at baseline as predictors. This linear regression model will allow correction for possible confounders like age, gender and working category.

- *Is there a difference between IBLA (5–12 weeks) and conventional ACL reconstruction?*

Results from study 2 will be used in a linear regression model with IKDC at 6 months as outcome and treatment, and IKDC at baseline as predictors. This linear regression model will allow correction for possible confounders like age, gender and working category.

- *Is there a difference between DIS and conventional ACL reconstruction?*

Results from study 1 and study 2 will be compared using a linear regression model with IKDC at 6 months as outcome and treatment, and IKDC at baseline as predictors. As this is a non-randomized comparison this model will correct for possible confounders like age, gender and working category.

For the primary analysis all patients that didn’t have a repairable, proximal ACL rupture will be excluded from the analysis (exploratory group). Patients with a failure or re-surgery will be included in the primary analysis.

#### Analysis of the secondary outcomes

- To evaluate robustness of the results a sensitivity analysis of the primary outcome will be done using different analysis sets.
- Analysis set without failures: patients that have a failure within the 6 months of follow-up are excluded from this analysis
- Analysis set without re-surgeries: patients that have a re-surgery within the 6 months of follow-up are excluded from this analysis
- In case we are not able to show superiority between the arms, we will consider non-inferiority between the arms. To this end we will choose 10 IKDC points as non-inferiority margin (see Hoogeslag et al. [25]). Non-inferiority will be considered in the different analysis sets and only be concluded if they give similar findings. The analysis sets considered here are intention-to-treat, the analysis set without failures and the analysis set without re-surgeries.
- As the IKDC scores are measured at many different time points during the patient’s follow-up we can compare the evolution over time between the treatment arms using a linear mixed model. Also here we will consider the effect of failure and re-surgery through inclusion and exclusion of the results after failure.
- Linear regression will be performed on the changes from baseline to 24 months in the continuous outcomes like VAS pain, VAS satisfaction, Tegner score, Lysholm score, EQ-5D-5 L, Lachman test, single leg and triple leg hop test, triple crossover hop and drop jump test, isokinetic and proprioceptive measurement and knee function. The evolution over time of these continuous outcomes will be compared between the different treatments using a linear mixed model.
- A survival analysis with log-rank test will be used to compare the time to return to work and time to return to sports between the different treatment arms. The time from the surgery until the patient is mobile without crutches will be analysed in the same way.
- The number of physiotherapy/orthopaedic surgeon consults will be compared between the different treatment arms using linear regression.
- The morphology and signal intensity of the ACL will be assessed with MRI and compared between the different treatments using a Chi-square test at the different time points.

In all the analyses there will be appropriately corrected for possible confounders (age, gender, working category, Tegner score of more than 7) especially in the analysis where the ACL reconstruction arm is compared to the DIS arm as this is a non-randomized comparison. As the sample size is small mainly ANCOVA models where only one covariate is added will be explored. As the sample size will not allow to combine all covariates in the model any conclusion for the non-randomized comparison will be carefully stated.

#### Analysis of safety endpoints

- The number of patients with SAEs will be reported per treatment arm.
- The proportion of patients having complications will be compared between the different treatments using a Chi-square test.

#### Exploratory analyses

- The IBLA arm (0–4 weeks) will be compared to the IBLA arm (5–12 weeks) to assess the optimal time frame for surgery.
- To identify if the patients that are intra-operatively confirmed of having an unrepairable and/or non-proximal, primary ACL rupture have certain characteristics in common and as such are an identifiable sub-population.

### Cost-utility analysis

The health economic evaluation will possibly demonstrate the improved economic impact on the healthcare system of the new ACL repair techniques compared to the conventional ACL reconstruction. In addition, a second analysis will possibly determine which of the two ACL repair techniques, DIS or IBLA, provides the best economic benefits also depending on the given clinical time frame. A cost-utility analysis will be used for the economic evaluations, since the main objective is the impact on HRQoL.

The result of the cost-utility analysis will be the Incremental Cost-Utility Ratio (ICUR) [33, 34]. In order to calculate the ICUR, we need to calculate the cost and the Quality-Adjusted Life Year (QALY), since ICUR = cost/QALY.

#### Cost

Healthcare costs need to be evaluated from the perspective of the healthcare payer. This includes payments out of the federal government’s and the communities’ healthcare budget as well as patients’ co-payments. The reference case analysis includes only the direct healthcare costs. These costs are directly related to the treatment of the disease (health services, hospitalization, etc.) as well as direct healthcare costs related to the disease in life years gained (e.g. occurring ADEs in the mid-term).

The productivity losses are indirect non-healthcare costs and will be presented in a separate, complementary analysis. These losses result from impairment of capacity to work. Short-term losses of productivity during paid work will be quantified by the human capital approach, i.e. the period-related income of the patient arm concerned. Productivity costs in the human capital approach are calculated by multiplying the total number of days of work absenteeism by the national average labour cost per day. Labour costs include employee wages and/or salaries and employer’s social security contributions. The Belgian average labour cost per working day is estimated at €257.

#### Utility

A cost-utility analysis includes a HRQoL measurement in the assessment of treatment outcome. One of the trial’s aims is to calculate the intervention’s cost-utility to support a reimbursement request. To measure the impact on HRQoL we will use the EQ-5D-5 L generic instrument, as recommended by the Belgian guidelines [33]. By using a generic utility instrument the comparability of the outcomes of these analyses may be improved. This generic instrument will be used to calculate the reference case and in a sub-analysis the disease specific instrument, the IKDC score, will complement this analysis.

The utility values which correspond to the EQ-5D-5 L health states range from 0 to 1, where 0 is the value of death and 1 is the value of perfect health. These index values (utility values) will be calculated by using the value set of United Kingdom, since the Belgian value set is not (yet) available. QALYs will be used as an outcome measure which combines HRQoL and survival. QALYs are preferred for cost-utility analysis because of their clarity, simplicity, ease of application, and face validity [33, 34]. Additionally, sensitivity analyses will be performed to examine the effect of uncertainty about the utility values.

In the final report the conclusions drawn in the clinical study analysis will be combined with the outcomes of the cost-utility analysis.

## Discussion

This LIBRƎ (Ligamys®, *Internal*Brace™, REconstruction) study protocol is the first to compare DIS, IBLA and the gold standard ACL reconstruction for relative clinical efficacy and economic benefit. In this 2-year follow-up study subjective, objective and functional outcomes will be compared in patients treated with DIS, IBLA or ACL reconstruction. The hypothesis of this study is that DIS and IBLA will prove to be more effective than the conventional ACL reconstruction 6 months post-operatively, based on the IKDC score.

There are several cohort studies on the short an mid long term result on ACL repair, providing a proof of concept and justifying further research. The theoretical advantages of primary ACL repair compared to reconstruction include preservation of the native ACL, its proprioceptive function, and avoidance of morbidity associated with graft harvesting. The preservation of the proprioceptors could attribute to a reduced recovery time and consequently a reduced need for costly physiotherapy, and faster return to work and sports [17, 20, 21]. So far, only one study has been published which compared DIS with the ACL reconstruction [25]. In contrast to the study of Hoogeslag et al. [25], our study is single-blind which means that the patients and the physiotherapists were blinded in order to reduce bias. In addition, we explicitly asked the patients to think about the pre-rupture situation when filling-in the pre-operative IKDC score so there would not be misinterpretation. Another strength of this study is the standardised rehabilitation protocol for the physiotherapists. Longitudinal MRI follow up after ACL repair could give additional information on the healing of the ACL and potential failures. The longitudinal isokinetic and proprioceptive measurements will give feedback on the rehabilitation period and the claim that by maintaining your own ACL, the proprioceptors are maintained as well [17]. In addition to the clinical effectiveness analysis, a cost-utility analysis will be performed to provide economic support for the most indicated surgery technique after an acute ACL rupture.

The ideal scenario would have been to include the ACL reconstruction group in Study 1 and defer the ALC reconstruction until 5 weeks post-injury, but this is a design choice we did not make for several reasons. The DIS technique has to be performed within 4 weeks post ACL rupture and ACL reconstruction after the knee has regained its function, often after 4 weeks post ACL rupture. IBLA can be performed up to 12 weeks post ACL rupture. If ACL reconstruction would be performed within the 4 weeks this would not be according to clinical practice. If the patients in Study 1 would have to be recruited in a 3 instead of 2 arms comparison, 78 patients would have to be recruited within the 4 weeks after ACL rupture. Due to patient and doctors referral delay, many of the patients with a ruptured ACL report to our orthopaedic departments after 4 weeks and therefore could not be included in the 3 arm comparison. We expected the early patient group (0–4 weeks) to be more challenging to recruit compared to the 5–12 weeks post rupture group; 78 patients within 0–4 weeks would substantially prolong the inclusion period. Patient blinding would be more difficult as patients can easier guess which treatment they received depending on the rupture-surgery time. For both the 0–4 and 5–12 weeks groups, after inclusion a date of surgery will be chosen, afterwards the randomization will be performed. If first the randomization will be performed and afterwards the date of surgery is chosen with the patients, it could give a clue which type of surgery the patient will receive. Hence, we decided to organize our study according to the principle: randomize each patient to the possible treatment options at the time of presentation post-rupture, corresponding with common clinical practice.

Ahmad et al. conclude in their systematic review paper that there is sufficient evidence to support that DIS repair may be an effective modality for the treatment of acute proximal tears of the ACL [48]. However, comparative studies are lacking. Upcoming studies should compare the technique to ACL reconstruction with failure as an endpoint. Comparison to rigid methods of proximal fixation is also necessary to justify the need for dynamic fixation. In the LIBRƎ study, the aforementioned points are compared. Overall, there is evidence to suggest the potential space for ACL repair in the decision tree for individualized treatment planning.

## Conclusions

This LIBRƎ study protocol is the first study to compare DIS, IBLA and ACL reconstruction for relative clinical efficacy and economic benefit. The outcomes of this study will provide data which could aid orthopaedic surgeons to choose between the different treatment options for the surgical treatment of an acute ACL rupture.

## Data Availability

Not applicable – this paper does not contain any data.

## Abbreviations

ACL: Anterior cruciate ligament
ADE: Adverse device effect
DIS: Dynamic intraligamentary stabilization
eCRF: electronic case report form
EDC: Electronic data capture
FWO: Fonds Wetenschappelijk onderzoek
HRQoL: Health-Related Quality of Life
IBLA: Internal brace ligament augmentation
ICUR: Incremental cost-utility ratio
IKDC: International knee documentation committee
MRI: Magnetic resonance imaging
QALY: Quality-Adjusted Life Year
RCT: Randomized controlled trial
SAE: Serious adverse event
VAS: Visual analogue scale
